# FSH mediates estradiol synthesis in hypoxic granulosa cells by activating glycolytic metabolism through the HIF-1α–AMPK–GLUT1 signaling pathway

**DOI:** 10.1016/j.jbc.2022.101830

**Published:** 2022-03-15

**Authors:** Gang Wu, Chengyu Li, Jingli Tao, Zhaojun Liu, Xiaoxuan Li, Ziyu Zang, Chen Fu, Jiayuan Wei, Yaxing Yang, Qian Zhu, Jia-Qing Zhang, Ming Shen, Honglin Liu

**Affiliations:** 1 College of Animal Science and Technology, Nanjing Agricultural University, Nanjing, China; 2 Institute of Animal Husbandry and Veterinary Science, Henan Academy of Agricultural Sciences, Zhengzhou, China

**Keywords:** hypoxia, granulosa cell, FSH, E2, glycolysis, 3β-HSD, 3β-hydroxysteroid dehydrogenase, AMPK, AMP-activated protein kinase, FSH, follicle-stimulating hormone, GCs, granulosa cells, HIF-1α, hypoxia-inducible factor-1α

## Abstract

Owing to the avascular environment within ovarian follicles, granulosa cells (GCs) are believed to live in a hypoxic niche. Follicle-stimulating hormone (FSH)-mediated steroidogenesis is crucial for normal growth and maturation of ovarian follicles, but it remains unclear how FSH stimulates estradiol (E2) synthesis under hypoxic conditions. Here, we aimed to explore whether FSH affects the ATP production required for estrogen synthesis from the perspective of glucose metabolism. It was observed that the levels of both E2 and HIF-1α were markedly increased in a dose-dependent manner in mouse ovarian GCs after the injection of FSH *in vivo*, indicating that hypoxia/HIF-1α may be relevant to FSH-induced E2 synthesis. By treating hypoxic GCs with FSH *in vitro*, we further revealed that the activation of the AMP-activated protein kinase (AMPK)–GLUT1 pathway, which in turn stimulates ATP generation, may be essential for FSH-mediated E2 production during hypoxia. In contrast, inhibition of AMPK or GLUT1 with siRNAs/antagonist both repressed glycolysis, ATP production, and E2 synthesis despite FSH treatment. Moreover, blocking HIF-1α activity using siRNAs/PX-478 suppressed AMPK activation, GLUT1 expression, and E2 levels in FSH-treated GCs. Finally, the *in vitro* findings were verified *in vivo*, which showed markedly increased AMPK activity, GLUT1 expression, glycolytic flux, ATP levels, and E2 concentrations in ovarian GCs following FSH injection. Taken together, these findings uncovered a novel mechanism for FSH-regulating E2 synthesis in hypoxic GCs by activating glycolytic metabolism through the HIF-1α–AMPK–GLUT1 pathway.

17β-estradiol (E2) is a major form of estrogen that is primarily produced in the ovaries under stimulation by follicle-stimulating hormone (FSH) at the follicular phase. On the basis of the two-cell theory, E2 synthesis is a multi-step process jointly completed by the granulosa cells (GCs) and theca cells. First, progesterone is synthesized from cholesterol *via* the steroidogenic acute regulatory protein (STAR), the cholesterol side-chain cleavage enzyme (P450scc), and 3β-hydroxysteroid dehydrogenase (3β-HSD) in both theca and GCs and is converted to androstenedione *via* steroid 17α-hydroxylase (P450c17) in theca cells. Androstenedione is then transported into GCs, where it is converted to E2 by aromatase (CYP19A1) and 17β-hydroxysteroid dehydrogenase (17β-HSD) (1). During E2 synthesis, FSH functions by binding to the FSH receptor, which activates G proteins on the cell membrane, enabling adenylate cyclase to catalyze the conversion of ATP to cAMP. cAMP in turn stimulates a protein kinase A–dependent activation of CYP19A1. In addition, the catalytic conversion of androstenedione to estradiol by CYP19A1 also requires the consumption of ATP (2). Therefore, ATP is indispensable for FSH-mediated E2 synthesis.

In mammalian ovaries, the follicular capillaries are restricted to the theca layers outside the basement membrane, while GCs and oocytes live in an environment without blood vessels (3). During follicular development, the growing distance from GCs to capillaries further limits the accessibility of blood-derived O_2_ to GCs, creating a hypoxic microenvironment within follicles (4). Recent studies indicated that FSH-induced follicular development is also associated with a progressively intensified hypoxic status in ovarian GCs (5). To cope with hypoxia, cells have developed a number of adaptive mechanisms mediated by hypoxia-inducible factor-1α (HIF-1α) to promote the transcription of genes involved in diverse processes, such as angiogenesis, energy metabolism, and cell proliferation (6). For instance, enhanced lactate production results from anaerobic glycolysis limiting the production of ATP despite sufficient glucose supply under hypoxia. HIF-1α induces the expression of enzymes in glycolytic pathway, as well as expression of the glucose transporters GLUT1 and GLUT3, which promote cellular uptake of glucose, thereby facilitating additional production of ATP to maintain the energy balance in hypoxic cells (7, 8). However, it remains to be elucidated whether glycolysis is relevant to FSH-mediated E2 synthesis in GCs under hypoxic conditions.

The AMP-activated protein kinase (AMPK) is a key regulator of cellular energy balance (9). In response to a lower energy state (higher ADP/ATP or AMP/ATP ratio), AMPK is activated (10) to upregulate pathways required for fatty acid oxidation (11), glycolysis (12), and mitochondrial homologous stability (13), leading to increased ATP production through phosphorylation of the mTORC1 complex, a key regulator of anabolic metabolism (14). Since hypoxia represents a type of stress that decreases cellular ATP supplies, activation of AMPK is commonly observed in hypoxic cells (15). However, current evidence does not indicate a direct action of AMPK during FSH-induced E2 synthesis in hypoxic GCs.

Herein, we aimed to further define the role and mechanism of the FSH-triggered response in the regulation of E2 synthesis in hypoxic GCs. Our findings suggested a primary role for glycolytic metabolism in promoting E2 synthesis through the FSH–HIF-1α–AMPK–GLUT1 pathway.

## Results

### The process of FSH-induced E2 synthesis is associated with HIF-1α accumulation in ovarian GCs

**Figure 1:**
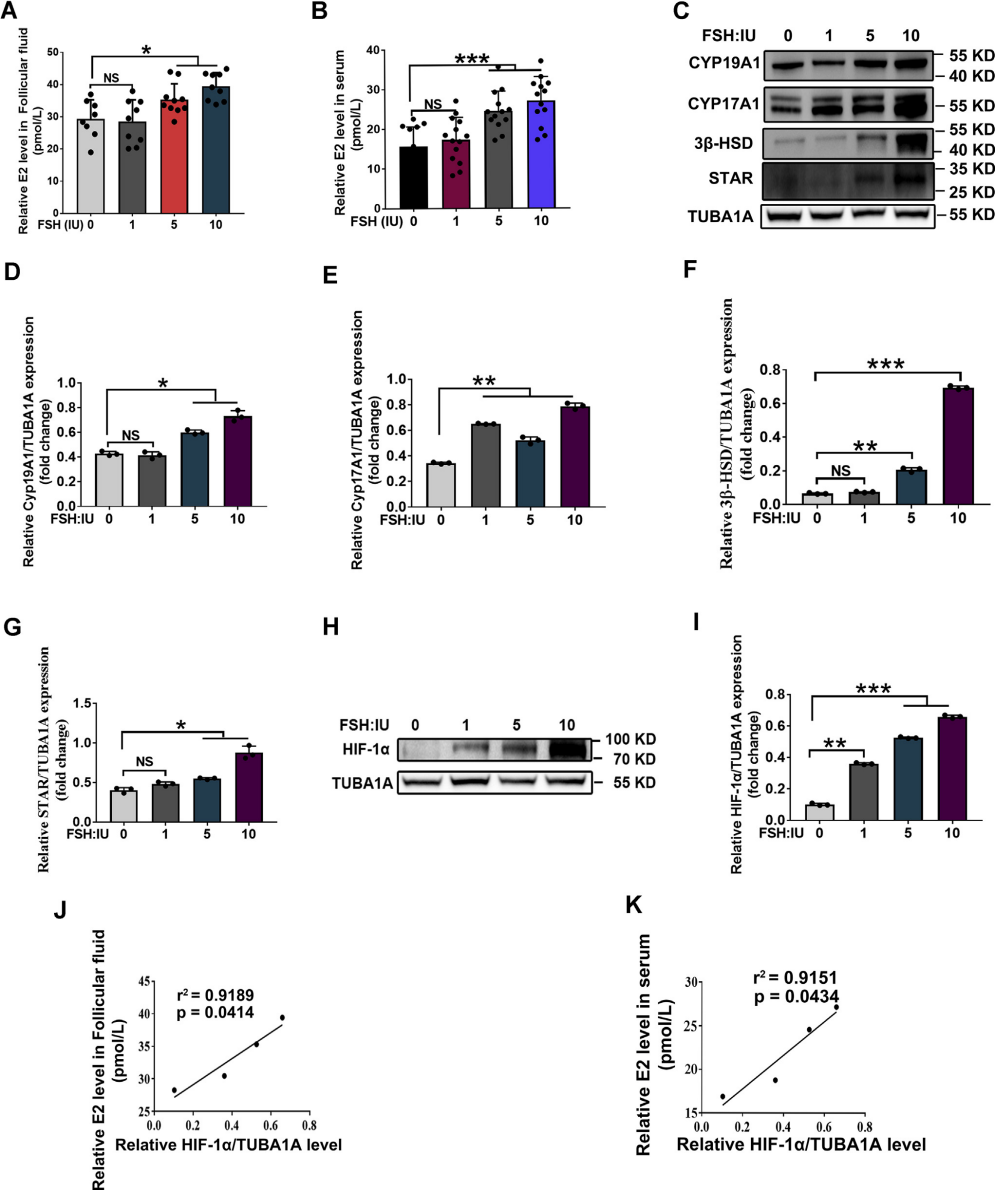
**FSH promotes E2 synthesis and HIF-1α accumulation in ovarian GCs.***A* and *B*, mice were injected intraperitoneally with 1 IU, 5 IU, and 10IU of FSH. The levels of E2 in follicular fluid (*A*) and serum (*B*) were examined by ELISA. *C*, effects of FSH on protein expression of steroidogenetic enzymes (including STAR, CYP17A1, CYP19A1, 3β-HSD) in ovarian GCs were examined by Western blot. *D*–*G*, quantitative analysis of protein levels in (*C*). TUBA1A served as the control for loading. *H*, effects of FSH on HIF-1α accumulation in ovarian GCs. *I*, quantitative analysis of HIF-1α level in (H). TUBA1A served as the control for loading. *J* and *K*, the relationship between E2 level in follicular fluid (*J*) or serum (*K*) and HIF-1α accumulation in ovarian GCs was analyzed by linear regression. The corresponding data above are represented as mean ± SD. ∗*p* < 0.05; ∗∗*p* < 0.01; ∗∗∗*p* < 0.001. 3β-HSD, 3β-hydroxysteroid dehydrogenase; FSH, follicle-stimulating hormone; GCs, granulosa cells; HIF-1α, hypoxia-inducible factor-1α; NS, not significant.

Using an ELISA assay, we determined the E2 levels in mice intraperitoneally injected with different concentrations of FSH. As shown in Figure 1, *A* and *B*, FSH caused a dose-dependent increase of E2 levels in both follicular fluid and serum. Consistent with this finding, protein expression levels of steroidogenic enzymes, including STAR, 3β-HSD, CYP19A1, and CYP17A1 were markedly upregulated after FSH injection (Fig. 1, *C*–*G*). We also detected the effects of FSH on the hypoxic response by examining HIF-1α expression in ovarian GCs. As shown in Figure 1, *H* and *I*, increased HIF-1α levels were observed in GCs collected from mice injected with FSH. Of note, linear regression analysis showed that the levels of E2 in follicular fluid and serum were positively correlated with HIF-1α content in ovarian GCs (Fig. 1, *J* and *K*). These data suggested the potential relevance of hypoxia/HIF-1α to FSH-mediated E2 synthesis.

### HIF-1α facilitates FSH-mediated E2 synthesis in GCs cultured under hypoxia

To further test whether hypoxia exerts any influence on FSH-induced E2 synthesis, as well as rule out interference from other follicular factors *in vivo*, we performed *in vitro* experiments using a previously established hypoxia model in porcine GCs. As shown in Fig. S1, the levels of HIF-1α protein displayed a time-dependent increase following hypoxia exposure. HIF-1α levels were further and markedly upregulated in hypoxic GCs receiving FSH treatment (Fig. S2).

**Figure 2:**
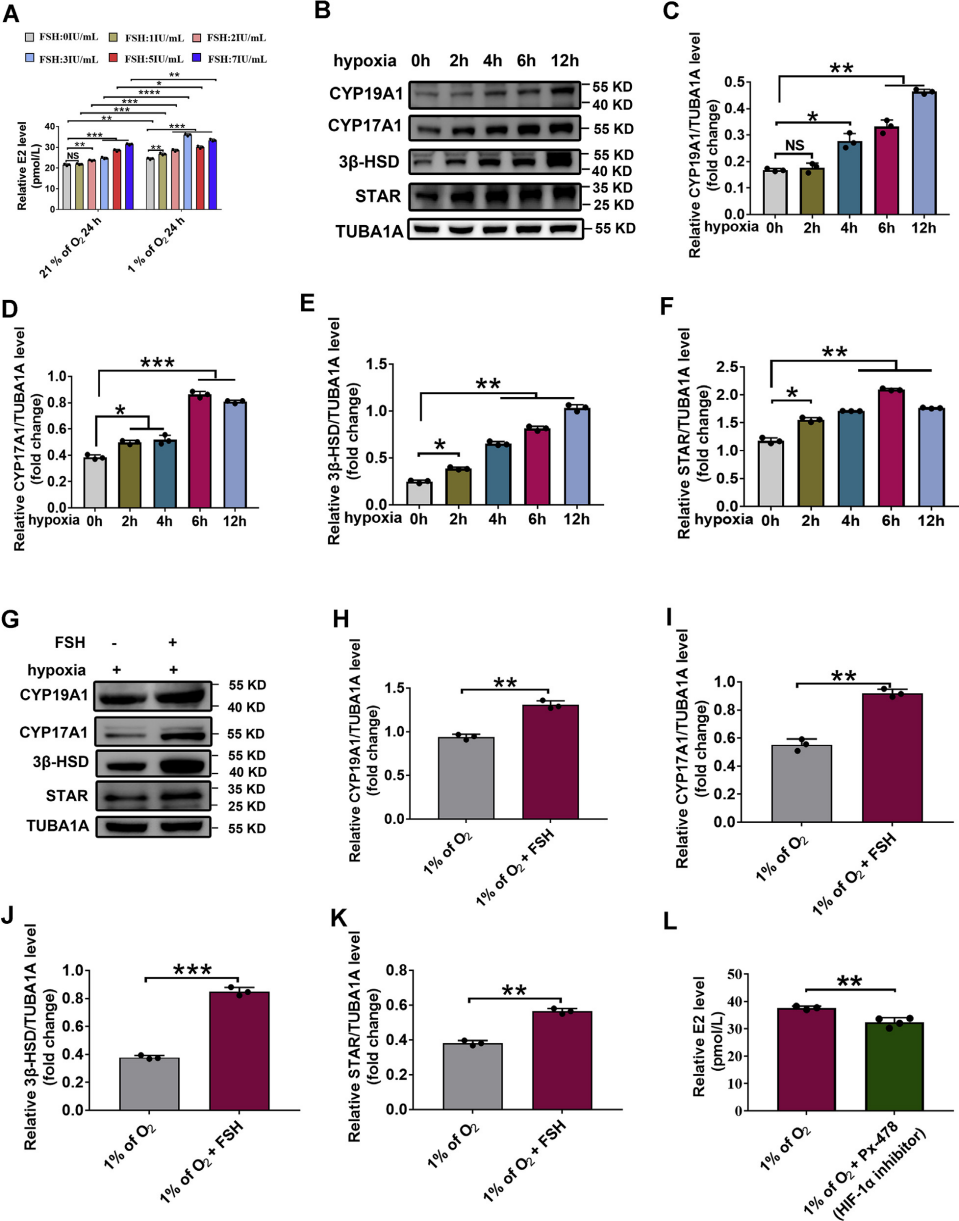
**FSH induces E2 synthesis in hypoxic GCs.***A*, primary porcine GCs were cultured under normoxia (21% O_2_) or hypoxia (1% O_2_) for 24 h in the presence or absence of FSH. The culture medium was then collected for examining E2 level using ELISA assay. *B*, GCs exposed to 1% of O_2_ for 0, 2, 4, 6, or 12 h were retrieved for Western blotting analysis of steroidogenetic enzymes as indicated. *C*–*F*, quantitative analysis of protein levels in (*B*). TUBA1A served as the control for loading. *G*, GCs cultured with hypoxia (1% of O_2_) and 3 IU/ml FSH for 24 h were collected for Western blotting analysis of steroidogenetic enzymes as indicated. *H* and *K*, quantitative analysis of protein levels in (*G*). *L*, GCs were cultured with hypoxia (1% of O_2_) and 3 IU/ml FSH for 24 h. For the inhibition of HIF-1α activity, PX-478 were added 2 h prior to hypoxia exposure. The culture medium was collected for examining E2 level using ELISA assay. The corresponding data above are represented as mean ± SD. ∗*p* < 0.05; ∗∗*p* < 0.01; ∗∗∗*p* < 0.001; ∗∗∗∗*p* < 0.0.01. FSH, follicle-stimulating hormone; GCs, granulosa cells; HIF-1α, hypoxia-inducible factor-1α; NS, not significant.

We next examined the levels of E2 in porcine GCs treated with different concentrations of FSH. As shown in Figs. S3*A* and 2*A*, significantly increased E2 levels were detected in hypoxic GCs following FSH treatment. In particular, cells treated with 3 IU/ml FSH exhibited the maximum production of E2 after 24 h of hypoxia exposure (Figs. 2*A* and S3, *B*–*D*). We applied this treatment protocol to the subsequent experiments. In accordance with E2 levels, GCs receiving FSH treatment also showed an elevated expression of steroidogenic enzymes (STAR, CYP19A1, CYP17A1, and 3β-HSD) during hypoxia incubation conditions (Fig. 2, *B*–*K*). Notably, compared with FSH-treated GCs in normoxia groups, significantly higher E2 levels were found in FSH-treated GCs under hypoxia (Fig. 2*A*). Using PX-478, an HIF-1α antagonist, we further investigated whether HIF-1α contributes to FSH-mediated E2 production. As shown in Figures. 2*L* and S6*E*, PX-478 treatment abolished the FSH-induced upregulation of E2 levels in hypoxic GCs. Collectively, these results suggested a possible involvement of HIF-1α in FSH-induced E2 synthesis during hypoxia.

### FSH enhances glycolytic flux in hypoxic GCs

**Figure 3:**
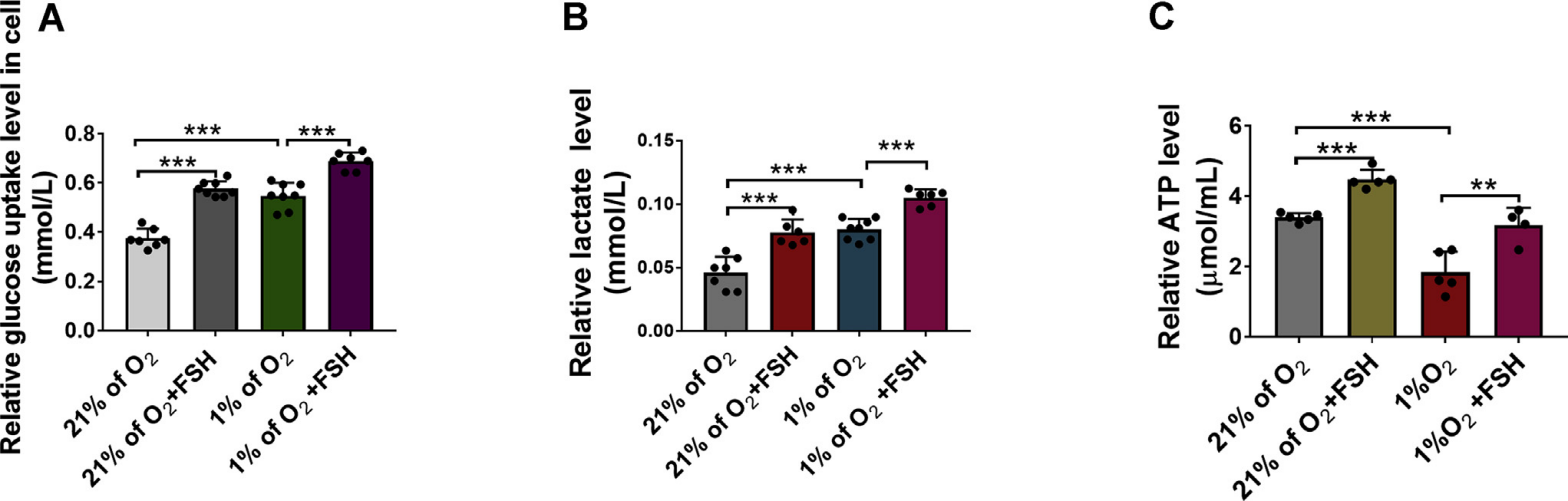
**FSH enhances glycolytic metabolism in hypoxic GCs.***A*, measurement of glucose levels in GCs with indicated treatments. *B*, determination of lactate levels in GCs with above-mentioned treatments. *C*, GCs were cultured under normoxia (21% O_2_) or hypoxia (1% O_2_) for 24 h in the presence or absence of FSH. Cells were then collected for determination of ATP levels. The corresponding data above are represented as mean ± SD. ∗∗*p* < 0.01; ∗∗∗*p* < 0.001. FSH, follicle-stimulating hormone; GCs, granulosa cells.

Estradiol synthesis has been described as an energy-consuming process (2). Under hypoxia, energy mainly comes from glycolysis metabolism. Therefore, we examined indicators related to glucose metabolism, including glucose uptake, lactic acid, and ATP production. We detected glucose uptake by measuring glucose absorption in GCs. As expected, the results showed that hypoxia significantly increased the level of glucose uptake, which was further upregulated in GCs treated with FSH (Fig. 3*A*). Moreover, FSH treatment significantly increased the lactate and ATP content in hypoxic GCs, reflecting an enhanced glycolytic flux (Fig. 3, *B* and *C*). Based on these findings, we speculated that FSH may promote E2 synthesis through glycolytic metabolism in GCs under hypoxia.

### Suppression of AMPK inhibits glycolytic metabolism and E2 production in FSH-treated GCs

**Figure 4:**
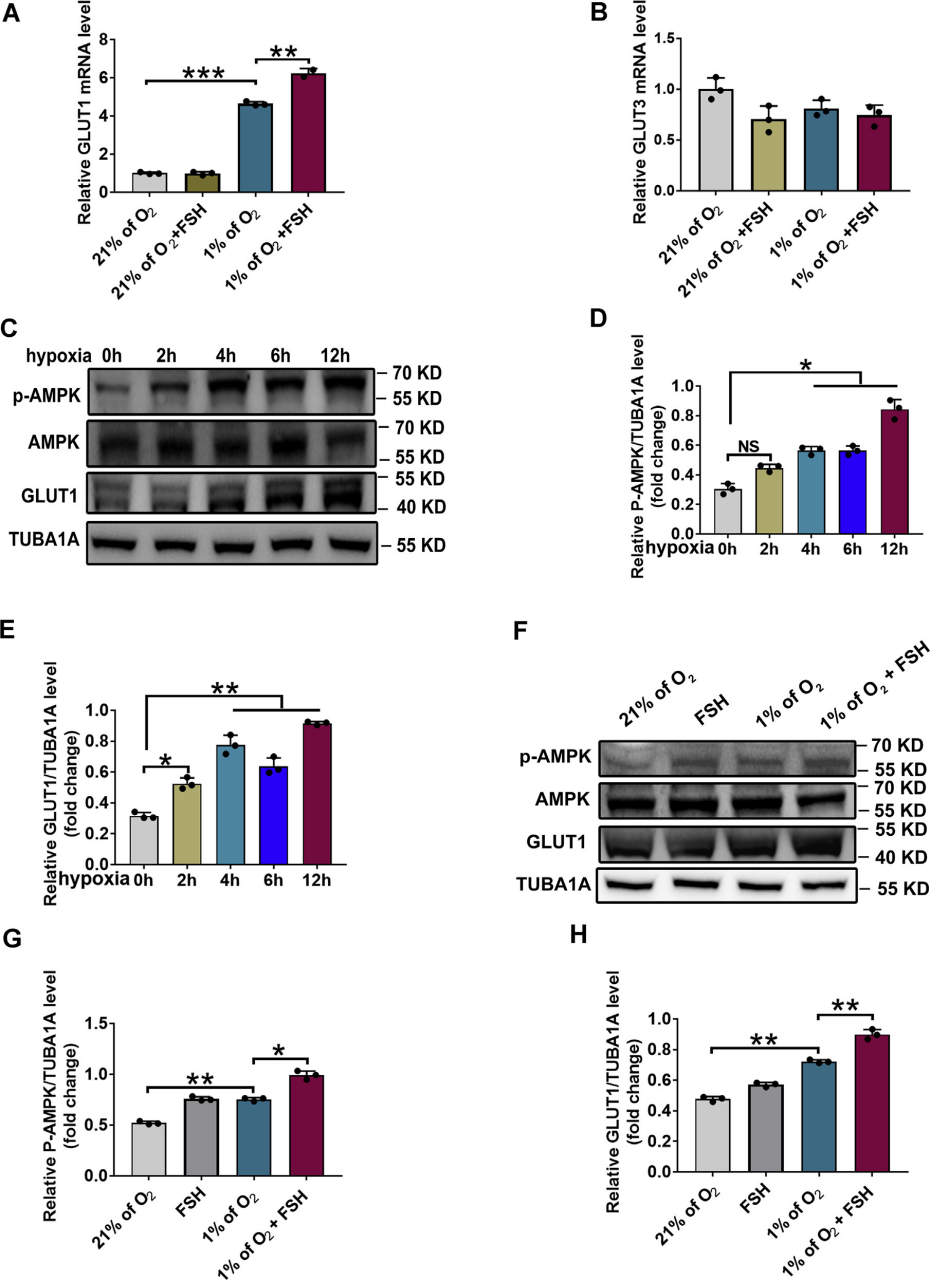
**FSH activates the AMPK/GLUT1 signaling in hypoxia.***A* and *B*, GCs were cultured under normoxia (21% O_2_) or hypoxia (1% O_2_) for 24 h in the presence or absence of FSH. Quantitative RT-PCR was then performed to measure the mRNA levels of *Glut1* and *Glut3* in GCs. *C*, GCs exposed to 1% of O_2_ for 0, 2, 4, 6, or 12 h were retrieved for Western blotting analysis of p-AMPK and GLUT1 levels. *D* and *E*, quantitative analysis of protein levels in (*C*). *F*, GCs were cultured under normoxia (21% O_2_) or hypoxia (1% O_2_) for 24 h in the presence or absence of FSH. Proteins levels of p-AMPK and GLUT1 were determined by Western blot. *G* and *H*, quantitative analysis of protein levels in (*F*). The corresponding data above are represented as mean ± SD. ∗*p* < 0.05; ∗∗*p* < 0.01; ∗∗∗*p* < 0.001. AMPK, AMP-activated protein kinase; FSH, follicle-stimulating hormone; GCs, granulosa cells; NS, not significant.

Previous reports demonstrated that AMPK, the key regulator of cellular energy balance, is activated by hypoxia to upregulate pathways required for glycolysis, as well as the expression of glucose transporters GLUT1 and GLUT3, which promote the cellular uptake of glucose (16). Therefore, we investigated the expression of GLUT1, GLUT3, and AMPK by qPCR and Western blot. The results showed that mRNA expression levels of *Glut1*, rather than *Glut3,* were significantly increased in hypoxic GCs after FSH treatment (Fig. 4, *A* and *B*). Likewise, the protein levels of GLUT1 exhibited a time-dependent increase following hypoxia incubation and was accompanied by activation of AMPK, as reflected by the elevated phosphorylation status at Thr172 (Fig. 4, *C*–*E*). Moreover, compared with hypoxia-treated GCs, the expression levels of GLUT1 and p-AMPK were markedly upregulated after FSH administration (Fig. 4, *F*–*H*).

**Figure 5:**
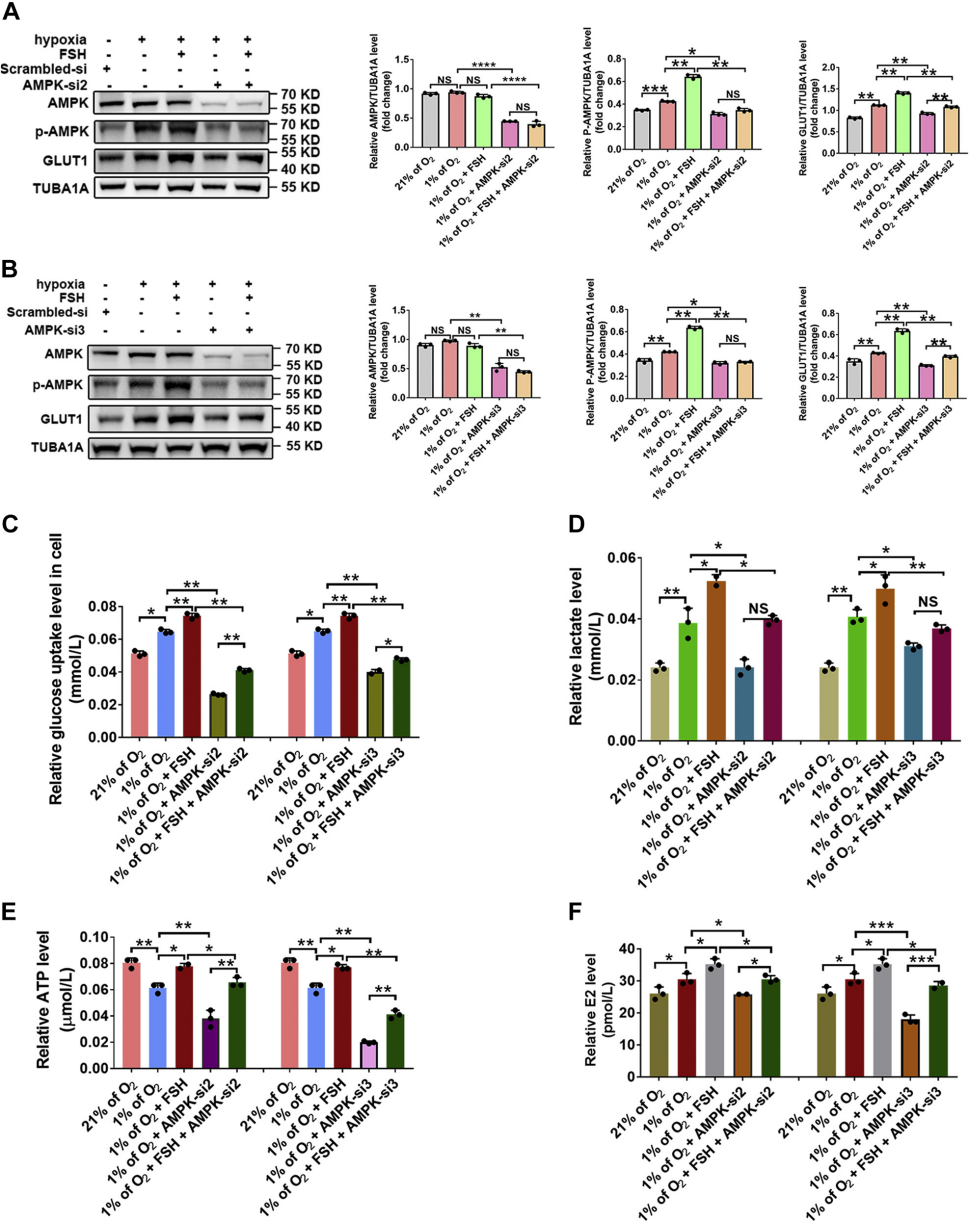
**Knockdown of AMPK inhibits glycolytic metabolism and E2 production in FSH-treated GCs**. *A*, GCs were cultured with normoxia hypoxia in the presence or absence of FSH for 24 h. GCs transfected with *AMPK* siRNA2 or scramble control siRNA for 12 h prior to hypoxia exposure. Proteins levels of p-AMPK and GLUT1 were determined by Western blot. *B*, GCs transfected with *AMPK* siRNA3 or scramble control siRNA for 12 h were cultured with or without 3 IU FSH for an additional 12 h under normoxia (20% of O_2_) or hypoxia (1% of O_2_) conditions. Proteins levels of p-AMPK and GLUT1 were determined by Western blot. *C*, glucose levels in GCs with indicated treatments. *D*, determination of lactate levels in GCs with indicated treatments. *E*, determination of ATP levels in GCs with indicated treatments. *F*, measurement of E2 levels in culture medium of GCs with treatments as mentioned above. The corresponding data above are represented as mean ± SD. ∗*p* < 0.05; ∗∗*p* < 0.01; ∗∗∗*p* < 0.001; ∗∗∗∗*p* < 0.0001. AMPK, AMP-activated protein kinase; FSH, follicle-stimulating hormone; GCs, granulosa cells; NS, not significant.

To further verify the impact of AMPK on E2 synthesis, we blocked AMPK activity by knocking down *AMPK* or using the inhibitor Compound C (COM.C). Three siRNAs against AMPK were first screened for their potency and specificity. Two of these candidates were identified as the most potent and specific siRNAs in silencing AMPK and used for subsequent experiments (Fig. S4*A*). Western blotting indicated that the expression of p-AMPK and GLUT1 upon FSH stimulation was inhibited after treatment with *AMPK* siRNAs or Compound C in hypoxic GCs (Figs. 5, *A* and *B*, S5, *A* and *B*). Consistent with these findings, blocking of AMPK activity augmented the cellular uptake of glucose (Figs. 5*C* and S5*C*) and was associated with the decreased production of lactate (Fig. 5*D* and S5*D*) and ATP (Fig. 5*E* and S5*E*). Moreover, FSH-induced upregulation of E2 synthesis was also abolished in hypoxic GCs after knocking down *AMPK* or using Compound C treatment (Fig. 5*F* and S5*F*). These data indicated that AMPK may contribute to FSH-mediated E2 synthesis by stimulating glycolytic metabolism in GCs exposed to hypoxic conditions.

### Knockdown of GLUT1 inhibits the effects of FSH on promoting glycolysis and E2 synthesis under hypoxia

**Figure 6:**
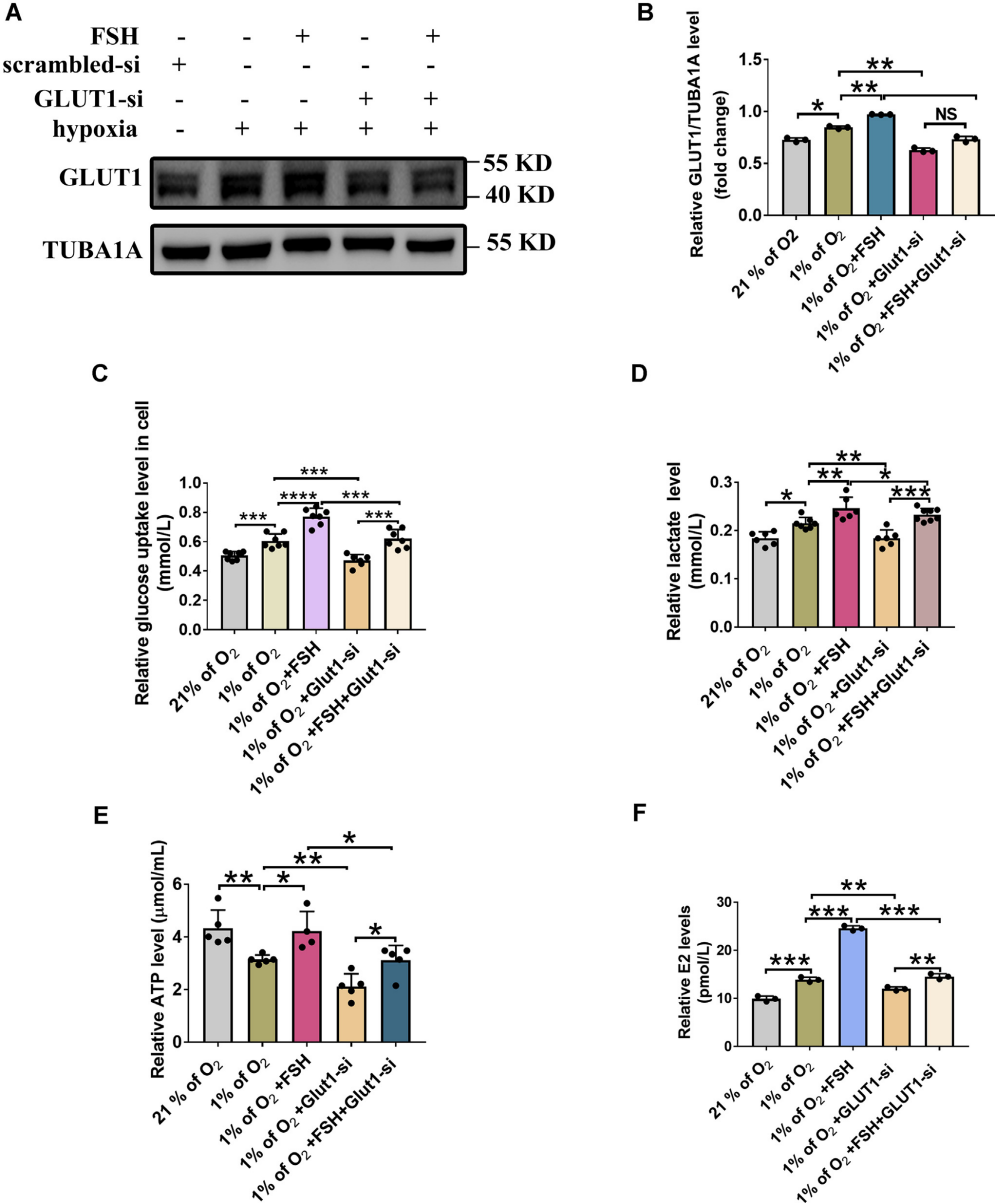
**Knockdown of GLUT1 inhibits the promotive effects of FSH on glycolytic metabolism and E2 production in hypoxic GC.***A*, GCs transfected with *Glut1* siRNA or scramble control siRNA for 12 h were cultured with or without 3 IU FSH for an additional 12 h under normoxia (20% of O_2_) or hypoxia (1% of O_2_) conditions. The protein level of GLUT1 was determined by Western blot. *B*, quantitative analysis of GLUT1 levels. TUBA1A served as the control for loading. *C*, glucose levels in GCs with indicated treatments. *D*, determination of lactate levels in GCs with indicated treatments. *E*, determination of ATP levels in GCs with indicated treatments. *F*, determination of E2 levels in culture medium of GCs with treatments as mentioned above. The corresponding data above are represented as mean ± SD. ∗*p* < 0.05; ∗∗*p* < 0.01; ∗∗∗*p* < 0.001; ∗∗∗∗*p* < 0.0001. FSH, follicle-stimulating hormone; GCs, granulosa cells; NS, not significant.

We next investigated the role of GLUT1 in FSH-mediated E2 synthesis. Specific siRNA targeting the transcripts of *Glut1* was designed to silence *Glut1* expression (Fig. 6, *A* and *B*). As shown in Figure 6, *C*–*F*, the glucose uptake in GCs (Fig. 6*C*) as well as the synthesis of lactate (Fig. 6*D*) and ATP (Fig. 6*E*) were significantly decreased after knocking down *Glut1* in FSH-treated GCs during hypoxia. In addition, the stimulatory effect of FSH on estrogen synthesis was also abrogated by *Glut1* siRNA in hypoxic GCs (Fig. 6*F*). Together, these results indicated that the AMPK–GLUT1 pathway may be required to mediate the action of FSH on glycolysis and E2 synthesis in GCs exposed to hypoxia.

### HIF-1α is required for activation of the AMPK–GLUT1 pathway and E2 synthesis in FSH-treated GCs

**Figure 7:**
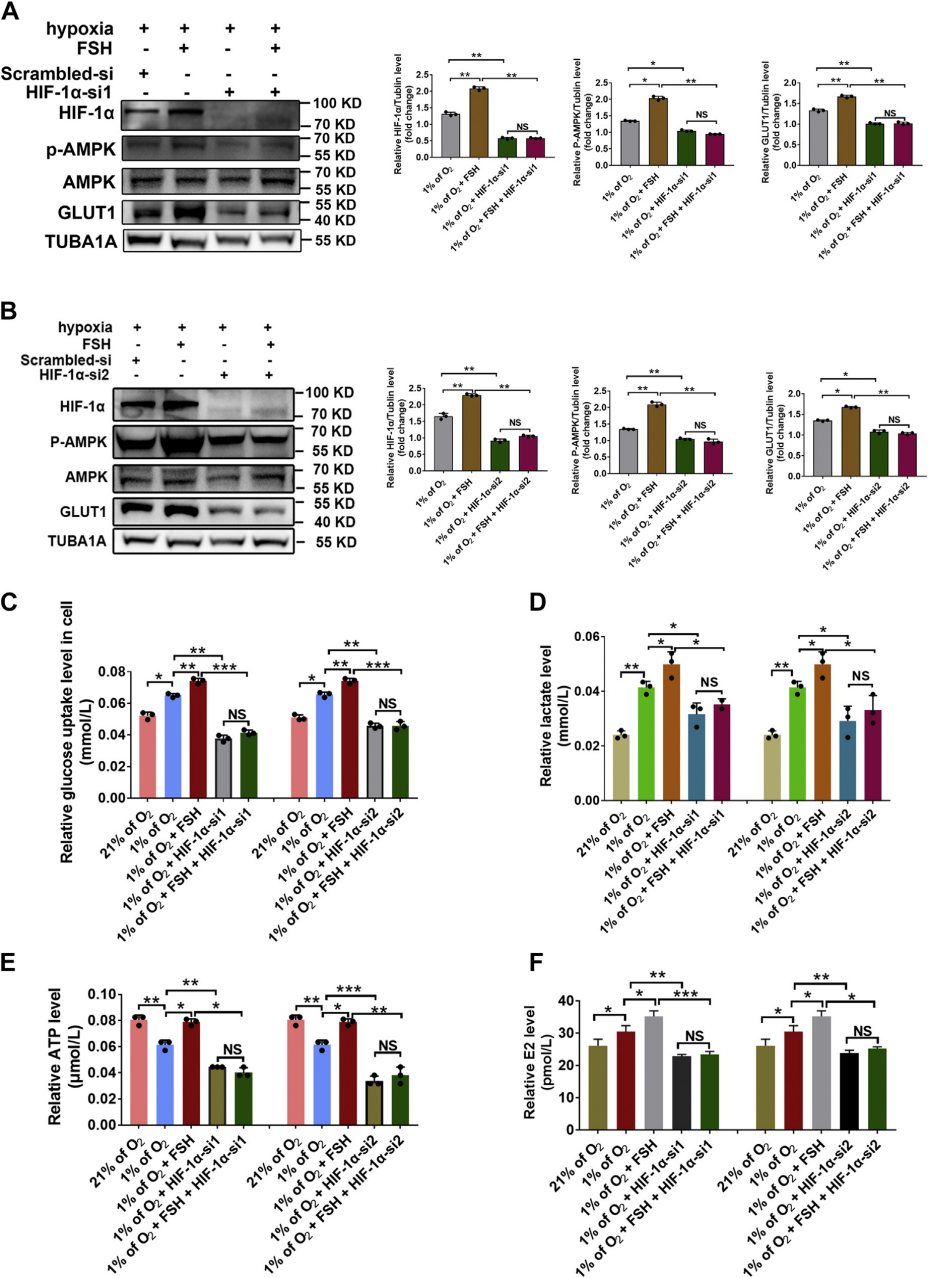
**FSH acts through HIF-1α to activate the AMPK–GLUT1 pathway in GCs exposed to hypoxia.***A*, GCs were cultured with hypoxia (1% of O_2_) and 3 IU/ml FSH for 24 h. GCs were transfected with *HIF-1α* siRNA1 or scramble control siRNA for 12 h prior to hypoxia exposure. Cell lysates were then collected for determining protein levels of p-AMPK and GLUT1 using Western blot. *B*, GCs transfected with *HIF-1α* siRNA2 or scramble control siRNA for 12 h were cultured with or without 3 IU FSH for an additional 12 h under normoxia (20% of O_2_) or hypoxia (1% of O_2_) conditions. Proteins levels of p-AMPK and GLUT1 were determined by Western blot. *C*, glucose levels in GCs with indicated treatments. *D*, determination of lactate levels in GCs with indicated treatments. *E*, determination of ATP levels in GCs with indicated treatments. *F*, determination of E2 levels in culture medium of GCs with treatments as mentioned above. The corresponding data above are represented as mean ± SD. ∗*p* < 0.05; ∗∗*p* < 0.01; ∗∗∗*p* < 0.001. AMPK, AMP-activated protein kinase; FSH, follicle-stimulating hormone; GCs, granulosa cells; HIF-1α, hypoxia-inducible factor-1α; NS, not significant.

To further clarify the relationship between HIF-1α and the AMPK–GLUT1 pathway in FSH-mediated E2 synthesis, hypoxic GCs were transfected with *HIF-1α* siRNA or treated with PX-478, an HIF-1α antagonist that was documented to inhibit HIF-1α at both transcriptional and translational levels (17). Three siRNAs against HIF-1α were first screened for their potency and specificity. Two of these candidates were identified as the most potent and specific siRNAs in silencing HIF-1α and used for subsequent experiments (Fig. S4*B*). As shown in Figures 7, *A* and *B*, S6*A*, both HIF-1α siRNAs and PX-478 markedly inhibited the expression of p-AMPK and GLUT1 induced by FSH treatment. Accordingly, glucose uptake in GCs (Figs. 7*C* and S6*B*) as well as the synthesis of lactate (Figs. 7*D* and S6*C*) and ATP (Figs. 7*E* and S6*D*) were significantly decreased after blocking HIF-1α activity in FSH-treated GCs during hypoxia. As shown in Figs. 7*F* and S6*E*, consistent results were obtained by determining E2 levels in GCs treated with HIF-1α siRNAs or PX-478, indicating that FSH may act through HIF-1α-dependent activation of the AMPK–GLUT1 pathway to induce E2 synthesis in hypoxic GCs.

### The *in vivo* validation of mechanisms for FSH-induced E2 synthesis in ovarian GCs

**Figure 8:**
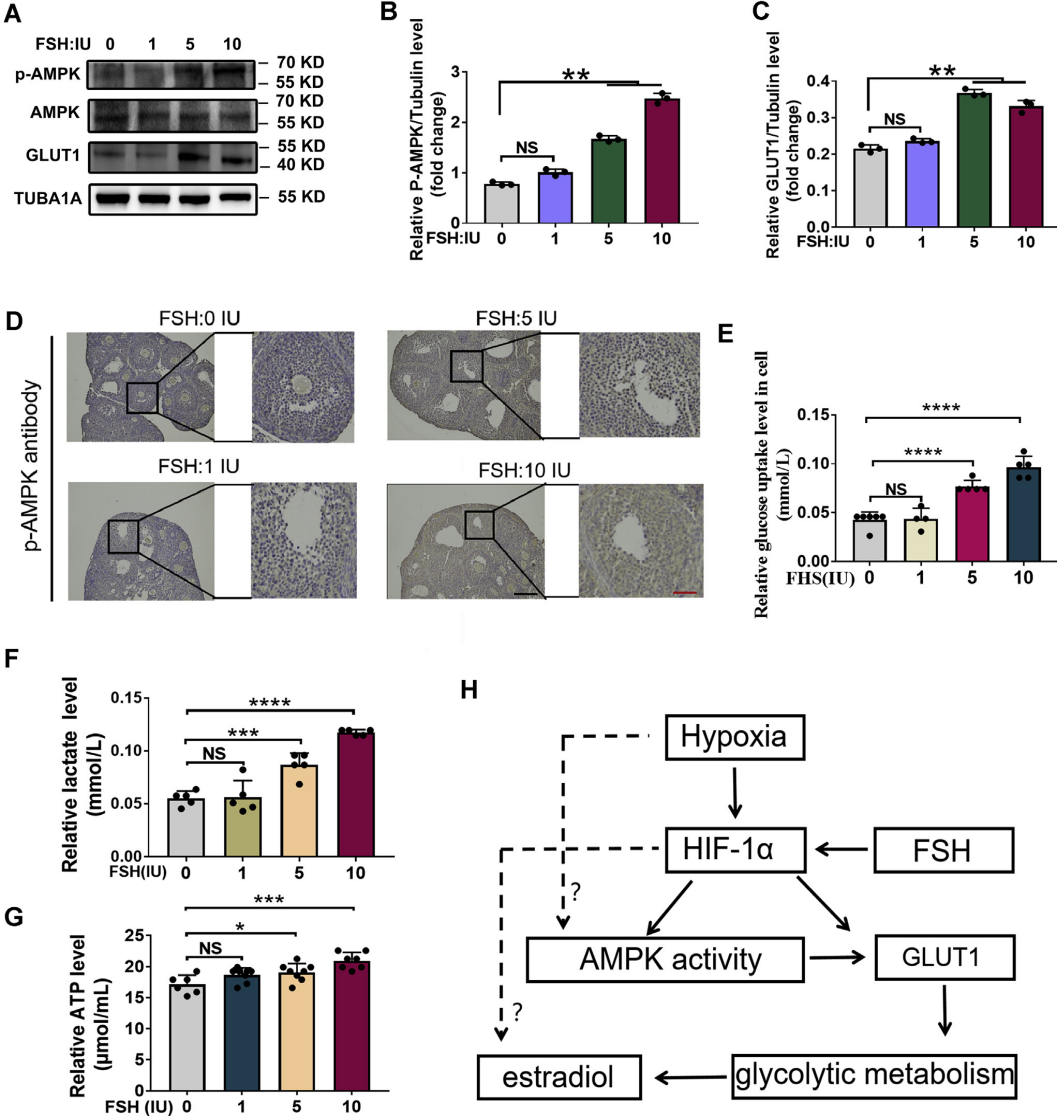
***In vivo* verification of the mechanistic model for FSH-mediated E2 synthesis in ovarian GCs.***A*, mice were injected intraperitoneally with 1 IU, 5 IU, and 10IU of FSH. The protein expression of GLUT1 and p-AMPK in ovarian GCs were examined by Western blot. *B* and *C*, quantitative analysis of protein levels in (*A*). *D*, the immunohistochemical staining of p-AMPK and hematoxylin in ovaries from FSH-injected mice. The scale bar represents 100 μm. *E*, glucose levels in ovarian GCs. *F*, lactate levels in ovarian GCs. *G*, ATP levels in ovarian GCs. *H*, schematic diagram showing FSH regulation of E2 synthesis in hypoxic GCs. The corresponding data above are represented as mean ± SD. ∗*p* < 0.05; ∗∗*p* < 0.01; ∗∗∗*p* < 0.001; ∗∗∗∗*p* < 0.0001. AMPK, AMP-activated protein kinase; FSH, follicle-stimulating hormone; GCs, granulosa cells; NS, not significant.

We next examined whether the *in vitro* mechanisms were applicable in ovarian GCs of mice injected with FSH. As shown in Figure 1, *H* and *I* and 8, *A*–*D*, the protein levels of HIF-1α, GLUT1, and phosphorylated AMPK were markedly upregulated in GCs following FSH treatment. Consistent with these findings, FSH injection increased the cellular uptake of glucose (Fig. 8*E*), which was accompanied by the elevated production of lactate (Fig. 8*F*) and ATP (Fig. 8*G*). Moreover, as shown in Figure 1, *A* and *B*, FSH administration triggered a dose-dependent increase of E2 levels in both follicular fluid and serum. Together, these findings provide *in vivo* evidence supporting the relevance of the HIF-1α–AMPK–GLUT1–glycolysis pathway to FSH-induced E2 synthesis.

## Discussion

In mammalian ovaries, FSH-mediated steroidogenesis plays an indispensable role in the recruitment, selection, and growth of ovarian follicles (18). FSH acts *via* its cognate FSH receptor that is expressed exclusively in GCs. In response to FSH stimulation, aromatase expression is activated to trigger anti-apoptotic/proliferative effects on GCs by promoting the synthesis of E2, a key sex steroid hormone required for follicular development and survival (19). Of note, FSH-induced follicular growth and development could also increase the distance between GCs and follicular capillaries, creating a progressively more hypoxic status within follicles (4). Yet, it remains undetermined whether hypoxia exerts any influence on FSH-mediated E2 synthesis. Using intraperitoneal injections of FSH, we observed a synchronized increase of both E2 synthesis and HIF-1α accumulation in mice ovarian GCs *in vivo* (Fig. 1). Cultured GCs receiving FSH treatment showed elevated E2 production and upregulated expression levels of steroidogenic enzymes (STAR, CYP19A1, CYP17A1, and 3β-HSD) during hypoxia exposure (Fig. 2). These data provide novel evidence, suggesting that hypoxia may be involved in the regulation of E2 synthesis mediated by FSH.

During episodes of compromised O_2_ availability, cells activate a number of adaptive responses to maintain cellular functions under hypoxia (20). Systemically, these adaptive mechanisms are coordinated by pathways involved in energy metabolism, angiogenesis, and cell proliferation under the regulation of HIF-1α, a commander of the cellular response to hypoxia (20). For instance, HIF-1α mediates the expression of multiple genes that promote angiogenesis/vascularization to increase oxygen supply in hypoxic tissues (21). As reported, HIF-1α also facilitates ATP generation in hypoxic cells by inducing the expression of genes involved in glycolysis and glucose uptake (22). Our previous studies confirmed that FSH promotes proliferation (23) and inhibits apoptosis (24) of follicular GCs by activating the HIF-1α-dependent hypoxia response, thus maintaining the development and survival of follicles under hypoxic conditions (25). The physiological effects of FSH on follicular development may be achieved in large part through the stimulation of E2 synthesis (26). In particular, during the transition from small growing follicles to large antral follicles, GCs acquiring FSH and E2 responsiveness begin to proliferate rapidly, initiating a developing phase with an exponential growth of follicular sizes (27). In this study, both *in vivo* and *in vitro* results showed a strong positive correlation between E2 levels and HIF-1α accumulation in GCs after FSH administration. Blocking HIF-1α activity diminished E2 production despite FSH treatment, indicating a requirement for HIF-1α in FSH-mediated E2 synthesis. These findings may contribute to clarify the functional mechanism of the hypoxia response pathway in FSH-induced follicular development and GCs proliferation.

Metabolic alterations in response to hypoxia trigger a direct switch from oxidative phosphorylation to glycolysis (28). Compared with oxidative phosphorylation, however, anaerobic glycolysis is significantly less efficient, providing a net production of only 2 ATP per glucose molecule (*versus* 32 ATP per glucose molecule produced during oxidative phosphorylation) (29). Since ATP serves as an essential energy substrate and signaling molecule in FSH-mediated E2 synthesis (2), hypoxia seems to be detrimental for this process. Interestingly, when GCs were cultured under hypoxia, we observed no decrease in E2 production in FSH-treated cells. This prompted us to investigate how FSH maintains the ATP supply required for E2 synthesis during hypoxia. Indeed, our current and previous data both showed a marked increase in HIF-1α levels in hypoxic GCs following FSH treatment (23). In terms of glucose metabolism, HIF-1α has been reported to promote the expression of key enzymes in the glycolysis pathway and to upregulate genes coding for glucose transporters, such as GLUT1 and GLUT3, enabling cells to uptake more glucose for glycolytic ATP production (30, 31). In accordance with these findings, our results showed that FSH acted through HIF-1α to induce the expression of GLUT1, which in turn increased the cellular uptake of glucose to facilitate glycolytic metabolism, thereby promoting ATP production and E2 synthesis in hypoxic GCs. These results suggest that FSH may satisfy ATP requirements for E2 synthesis under hypoxic conditions by activating the glycolysis pathway.

Notably, GCs that received 24 h of hypoxia exposure produced higher levels of E2 and lower levels of ATP compared with that in GCs cultured under normoxia conditions. After treatment with 3 IU FSH, E2 production in hypoxic GCs was 1.45 times higher than that in normoxic GCs, while ATP production did not reach the corresponding level found in normoxic GCs. These data indicate the existence of an ATP-independent mechanism for E2 synthesis under hypoxia. It is possible that HIF-1α could directly promote the synthesis of E2. Nevertheless, how hypoxia/HIF-1α influences E2 synthesis remains to be investigated in future studies.

AMPK is an evolutionarily conserved serine/threonine protein kinase known to be activated in response to a decreased AMP:ATP ratio (10). Under hypoxic conditions, AMPK induces ATP production in cells *via* a switch from anabolic to catabolic metabolism through the stimulation of glycolysis flux by increasing GLUTs-dependent glucose uptake (12). However, the relationship between AMPK and HIF-1α remains obscure. It has been documented that AMPK could cooperate with HIF-1α to promote the expression of GLUTs and an Na^+^ coupled glucose transporter (SGLT1) (32). There are also reports showing no crosstalk but antagonism between AMPK and HIF-1α in cancer cells (33). According to our previous study, FSH-induced accumulation of HIF-1α was accompanied by an elevation of AMPK activity in ovarian GCs (23). Herein, we observed downregulated expression levels of both p-AMPK and GLUT1 after blocking HIF-1α activity in FSH-treated GCs (Figs. 7, *A* and *B*, S6*A*). Additionally, inhibition of AMPK activity abrogated FSH-induced GLUT1 expression in hypoxic GCs (Figs. 5, *A* and *B*, S5*A*). Collectively, this work may provide the first evidence linking the HIF-1α–AMPK–GLUT1–glycolysis–ATP axis to FSH-mediated E2 synthesis under hypoxia.

A hypothetical model showing FSH-induced regulation of E2 synthesis in hypoxic GCs is shown in Figure 8*H*. The low oxygen environment within ovarian follicles causes the accumulation of HIF-1α in GCs. The presence of FSH induces a further increase of HIF-1α levels in hypoxic GCs. HIF-1α activates AMPK-dependent expression of GLUT1, resulting in accelerated uptake of glucose by GCs, thus facilitating glycolytic metabolism, which in turn promote the synthesis of E2. HIF-1α may directly upregulate GLUT1 expression to stimulate glycolysis flux and E2 synthesis. Moreover, hypoxia/HIF-1α may promote the synthesis of E2 through a glycolysis-independent mechanism.

## Experimental procedures

### Reagents and antibodies

Compound C and Px-478 were purchased from Selleck Chemicals. FSH was obtained from Ningbo Second Hormone Factory. Antibodies against AMPK (Cat. No. AF-6423) and p-AMPK (Cat. No. AF-3423) were purchased from Affinity Biosciences. GLUT1 antibody (Cat. No. 21829-1-AP) was purchased from Proteintech Group. Antibodies against CYP19A1 (Cat. No. A1336), CYP17A1 (Cat. No. A1337), 3β-HSD (Cat. No. A1823), and STAR (Cat. No. A16432) were purchased from ABclonal Technology.

### Animals and sample collection

All the animal experiments were conducted in accordance with the guidelines for the Care and Use of Animals in Research and Teaching, and the protocols were approved by the Committee of Animal Research Institute, Nanjing Agricultural University. Three-to-four-week-old female ICR mice (Qing Long Shan Co, Animal Breeding Center) were housed five per cage in a temperature-controlled (22 ± 2 °C) room with a 12:12 h light:dark cycle and had ad libitum access to water and food. The mice were randomly divided into control group and FSH group. Thirteen mice were included in each group. Each mouse in the treatment group was intraperitoneally injected with 1, 5, or 10 IU FSH (dissolved in 0.9% saline) every 12 h. The control mice were concomitantly injected with the same volume of 0.9% saline. Seventy-two hours after the first FSH injection, mice in each group were sacrificed for the collection of serum, follicular fluid, and GCs respectively.

### Cell culture and treatments

The *in vitro* study was performed using a previously established hypoxia model in porcine GCs (24). The isolation and culture of primary porcine GC culture were performed as described (34). Briefly, ovaries from mature sows collected at a local slaughterhouse (Changzhou Erhualian Pig Production Cooperation) were transferred to the laboratory immediately in 0.9% saline maintained at 37 °C. After washing three times with 0.9% saline, one time with 75% alcohol, and three times with 0.9% saline again, the ovaries were subjected to GCs aspiration from antral follicles using a syringe with a 20-gauge needle. GCs were then washed with PBS, centrifuged at 1000*g* for 5 min, and harvested for cell culture in a DMEM/F-12 (1:1) medium supplemented with 10% fetal bovine serum, 100 units/ml penicillin, 100 μg/ml streptomycin, and 25 mM Hepes for 1.5 days. In control group, GCs were maintained at 37 °C in a 5% CO_2_, 95% air incubator (21% of O_2_). Hypoxic cells (1% of O_2_) were cultured in a modular incubator chamber flushed with a gas mixture containing 1% of O_2_, 5% CO_2_, and 94% N_2_ at 37 °C. In each experiment, we used ovaries obtained from 5 sows, and a total of 10 ovaries were collected to obtain pooled GCs. These GCs were equally divided into control and experimental groups. For drug administration, GCs were cultured under normoxia (21% O_2_) or hypoxia (1% O_2_) in the presence or absence of FSH for the time as indicated. In some experiments, cells were treated with Compound C (AMPK inhibitor) or PX-478 (HIF-1α inhibitor) for 2 h prior to hypoxia exposure. For RNA interference, GCs were transfected with *Glut1* siRNA or scrambled control siRNA for 12 h and then cultured under normoxia or hypoxia for 12 h.

### Immunohistochemistry

The ovaries were fixed with 4% paraformaldehyde, dehydrated with gradient alcohol solutions (5%-100%), embedded in paraffin, serially sectioned to 5 μm with a microtome, and mounted on glass slides. After deparaffinization and rehydration, sections were subjected to antigen unmasking by microwave heating of the tissue in citrate buffer for 0.5 h. After 10 min of H_2_O_2_ incubation to quench intracellular peroxidase activity, ovarian sections were blocked in 1% bovine serum albumin and sequentially incubated with p-AMPK antibody (diluted 1:200) and biotin-labeled secondary antibody. The immunoreactive signals were visualized using the 3, 3′-diaminobenzidine chromogen solution. The nuclei were counterstained with hematoxylin before dehydration. Representative images were captured under a virtual microscope (Olympus).

### RNA interference

siRNAs specific for *Glut1, AMPK, HIF-1α,* and the scrambled control siRNAs (see Table S1 for siRNA sequences) were obtained from GenePharma. Transfection of siRNA was performed using Lipofectamine 3000 (Invitrogen) according to the manufacturer's instructions.

### Quantitative RT-PCR

Total RNA was isolated with TRIzol (Invitrogen) and reverse-transcribed into cDNA using PrimeScriptTM RT Master Mix (Takara). Quantitative RT-PCR was performed with Hieff UNICON qPCR SYBR Green Master Mix (Vazyme) and gene specific primers (see Table S2 for primer sequences) on the ABI StepOne PCR system (Applied Biosystems). The specificity of each PCR amplification was verified by melting curve analysis, and the data were normalized to the amount of *Tuba1a* expressed.

### Western blot

Cells were harvested in RIPA lysis buffer (Beyotime), and protein was quantified with the bicinchoninic acid assay (Beyotime). An equal amount of total protein (15 μg) was separated by PAGE and transferred to PVDF membrane by electroblotting. The nonspecific binding sites were blocked with Tris Buffered Saline with Tween 20 containing 5% bovine serum albumin for 1 h. Membranes were then probed with primary antibodies against AMPK, p-AMPK, GLUT1, STAR, CYP19A1, CYP17A1, or 3β-HSD overnight at 4 °C. After washing with Tris Buffered Saline with Tween 20, membranes were incubated with horseradish peroxidase–conjugated secondary antibody. Bands were visualized using WesternBright ECL HRP substrate kit (Advansta) according to the manufacturer’s directions.

### ELISA assay of E2 content

The level of E2 was determined using an E2 ELISA kit (Nanjing Aoqing Biological Technology Co, Ltd) following the manufacturer's instructions. Briefly, the samples of follicular fluid, serum, or culture medium were centrifuged at 2000*g* for 20 min. The supernatant resolved in the dilution buffer were added to the microelisa stripplate precoated with an antibody specific to E2, followed by 30 min of incubation at 37 °C with a horseradish peroxidase–conjugated antibody against E2. After washing, the substrate solution was added to trigger the chromogenic reaction. The absorbance was measured at 450 nm using a TECAN microplate reader. The concentration of E2 was calculated from the standard curve.

### Determination of glucose uptake and consumption

Glucose uptake was measured using a 2-Deoxyglucose Glucose Uptake Assay Kit (ab136955, Abcam) based on the protocol provided by the manufacturer. Briefly, the glucose analog 2-deoxyglucose was added to the cells and the accumulated 2-DG6P was oxidized to generate NADPH, which resulted in the oxidation of a substrate. The glucose concentration in tested samples was then evaluated by measuring OD values at a absorbance of 412 nm under a TECAN microplate reader.

### Determination of lactate production

The levels of lactate in GCs were determined by a lactate content detection kit (Beijing Solarbio Science & Technology Co, Ltd) according to the manufacturer’s instructions. The concentration of chromogenic products was measured at an absorbance of 570 nm using a TECAN microplate reader. Experiments were conducted in triplicate, and the results were normalized to the protein concentration of each sample (Beyotime Institute of Biotechnology).

### Measurement of ATP generation

The levels of ATP in GCs were determined with an ATP content detection kit (Beijing Solarbio Science & Technology Co, Ltd) following the manufacture’s protocols. Briefly, cell lysates were centrifuged at 10,000*g* at 4 °C for 10 min, and the supernatant was incubated in the ATP-detection buffer to generate the chromogenic products. The OD values were measured at an absorbance of 340 nm using a TECAN microplate reader. Experiments were performed in triplicate, and the ATP levels were normalized against protein concentration of each sample.

### Statistical analysis

Statistical analysis was performed using Prism 7 GraphPad Software. Data are expressed as mean ± SD. All experiments were repeated at least three times. Differences between groups were assessed using ANOVA, followed by LSD post hoc test. Values of *p* < 0.05 were considered significant.

## Conclusion

In summary, this study describes a novel model of FSH-mediated E2 synthesis in hypoxic GCs *via* the activation of glycolysis through the HIF-1α––AMPK–GLUT1 signaling pathway. Our findings provide new understanding of the responsive/adaptive mechanism of ovarian follicles to hypoxic conditions and also provide a theoretical basis for developing protocols used in improving animal reproduction by promoting E2 synthesis.

## Data availability

All data described are contained within this article and its Supporting information files.

## Supporting information

This article contains supporting information.

## Conflict of interest

The authors declare that they have no conflicts of interest with the contents of this article.
