# Supplementary material for: FSH mediates estradiol synthesis in hypoxic granulosa cells by activating glycolytic metabolism through the HIF-1α–AMPK–GLUT1 signaling pathway
Source: J Biol Chem. 2022 Mar 15;298(5):101830. doi: 10.1016/j.jbc.2022.101830 (PMC9036125; doi:10.1016/j.jbc.2022.101830)
Supplement: Supplemental Figures S1–S6, Tables S1 and S2 [file mmc1.docx]

**Supplementary Figure**

**
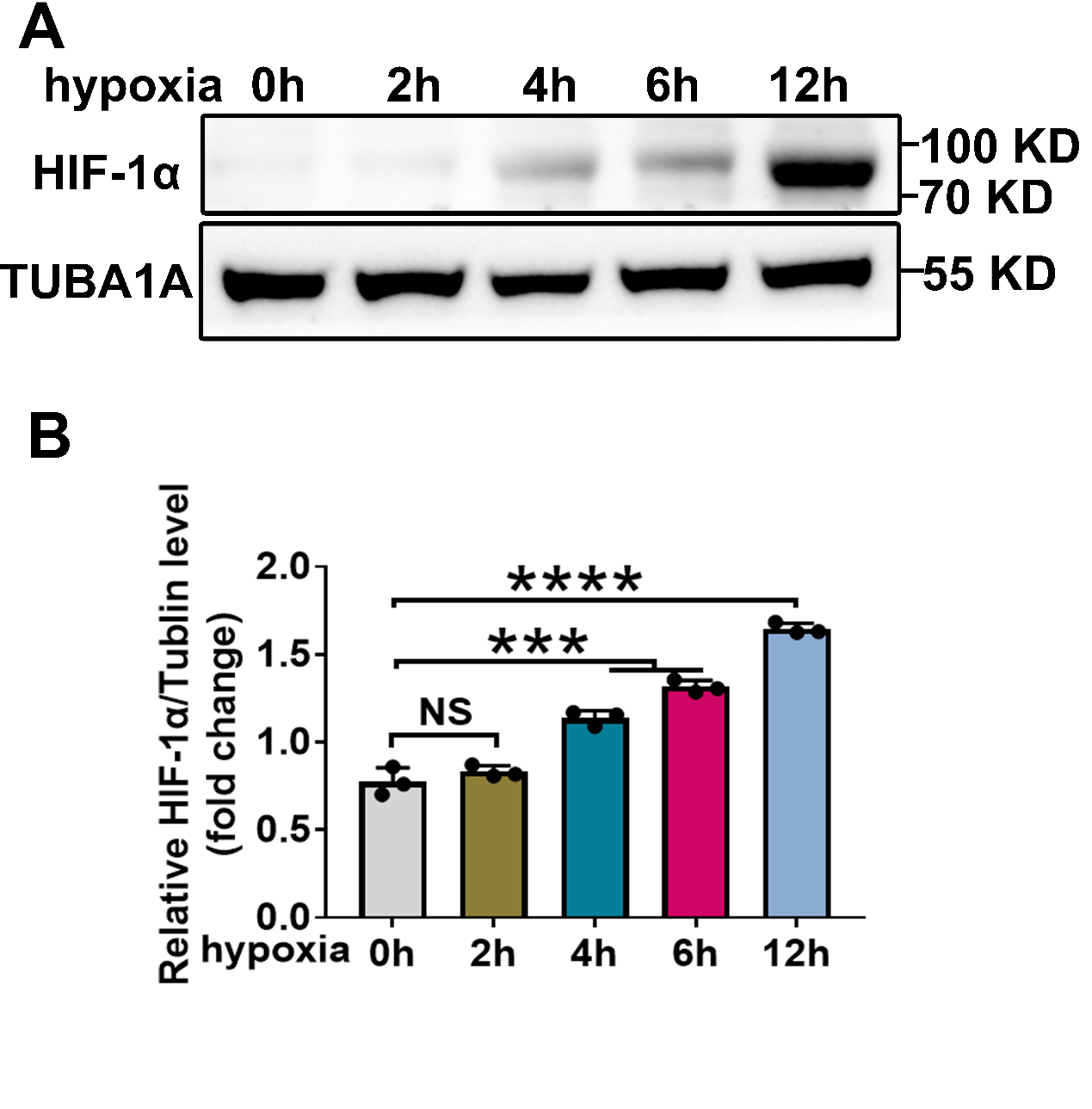
**

**Fig. S1**. HIF-1α expression is up-regulated in a time-dependent manner during hypoxia exposure. **A**. GCs exposed to 1% of O_2_ for 0, 2, 4, 6, or 12 h were retrieved for western blotting analysis of HIF-1α expression. **B**. Quantitative analysis of protein levels in (**A**). TUBA1A served as the control for loading. Data represent mean ± SD. *P < 0.05; **P < 0.01.


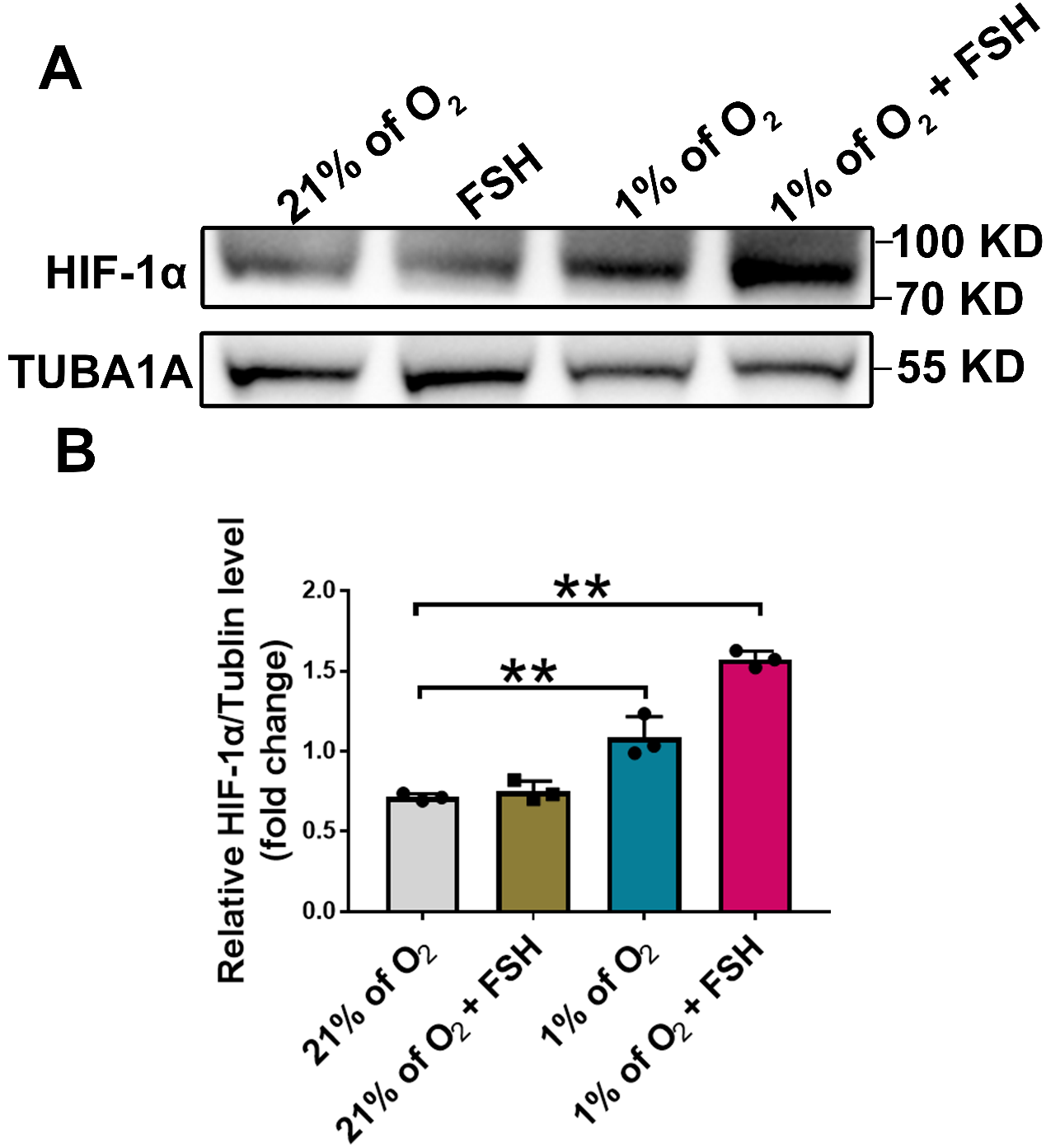


**Fig. S2.** HIF-1α protein level was up-regulated under hypoxia and further increased after FSH treatment. Primary porcine GCs were cultured under normoxia (21% O_2_) or hypoxia (1% O_2_) for 12 h in the presence or absence of FSH. GCs were then retrieved for western blotting analysis of HIF-1α levels. **B**. Quantitative analysis of HIF-1α levels in (**A**). TUBA1A served as the control for loading. Data represent mean ± SD. *P < 0.05; **P < 0.01; ***P < 0.001; ****P < 0.001.


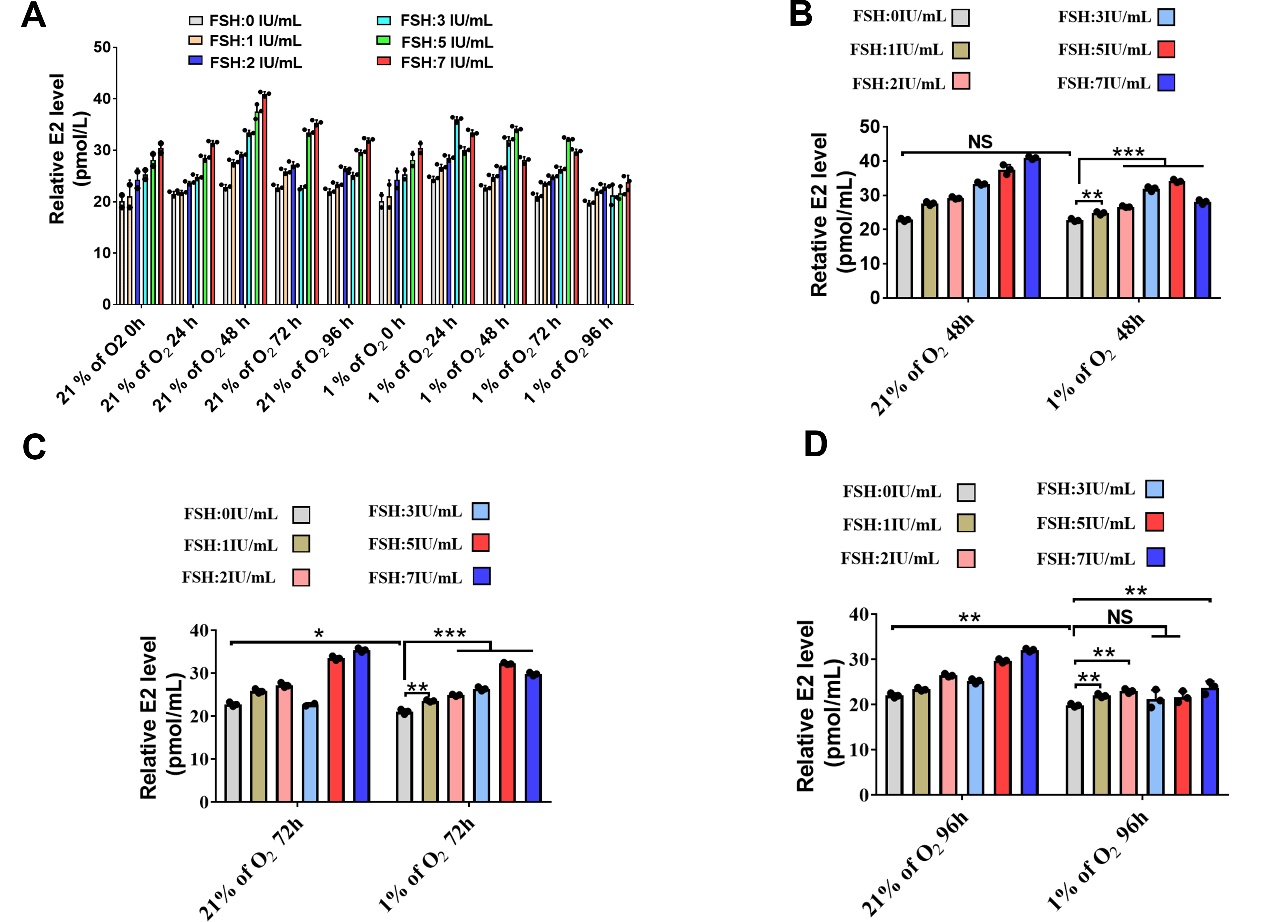


**Fig. S3.** Determination of E2 levels in FSH-treated GCs during hypoxia exposure. **A**. Primary porcine GCs were cultured with FSH (at concentrations ranged from 0-7 IU/ml) under normoxia (21% O_2_) or hypoxia (1% O_2_) for 0, 24, 48, 72, or 96 h. The culture medium was then collected for examining E2 level using ELISA assay. **B-D**. Statistical analysis of E2 levels in culture medium at 48 h (**B**), 72 h (**C**), and 96 h (**D**) after the indicated treatments. The corresponding data above are represented as mean ± SD. *P < 0.05; **P < 0.01; ***P < 0.001; NS, not significant, P > 0.05.


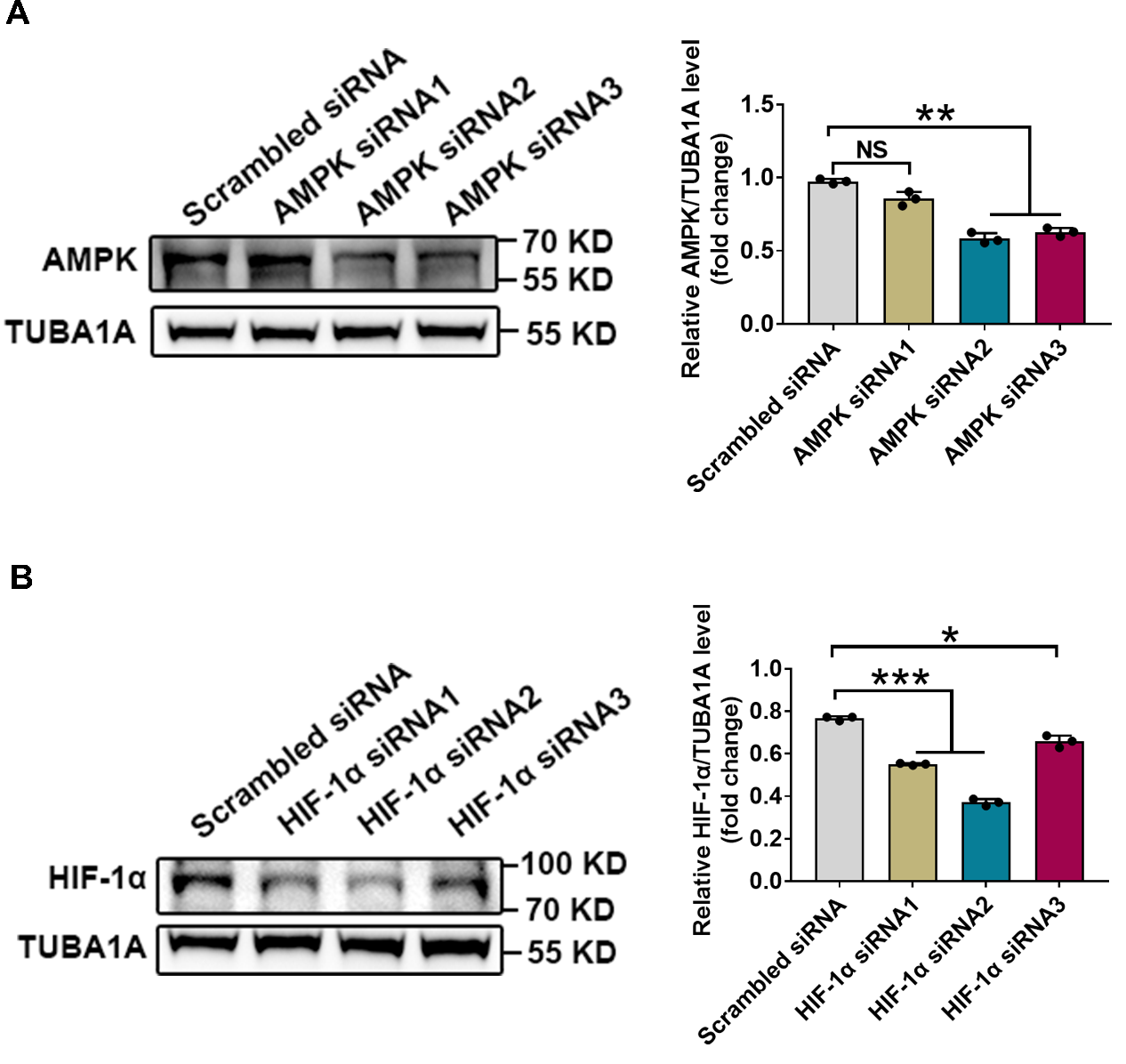


**Fig. S4** The gene silencing efficiency of siRNAs against AMPK and HIF-1α. **A, B**. Primary cultured GCs were transfected with AMPK siRNAs, HIF-1α siRNAs, or scrambled control siRNA for 12 h, and then collected for western blotting detection of the protein levels of AMPK (**A**) and HIF-1α (**B**) respectively. Two out of the candidate siRNAs in each group were identified as the most potent and specific siRNAs, and were used for the subsequent experiments. TUBA1A served as the control for loading. The corresponding data above are represented as mean ± SD. *P < 0.05; **P < 0.01; ***P < 0.001; NS, not significant, P > 0.05.


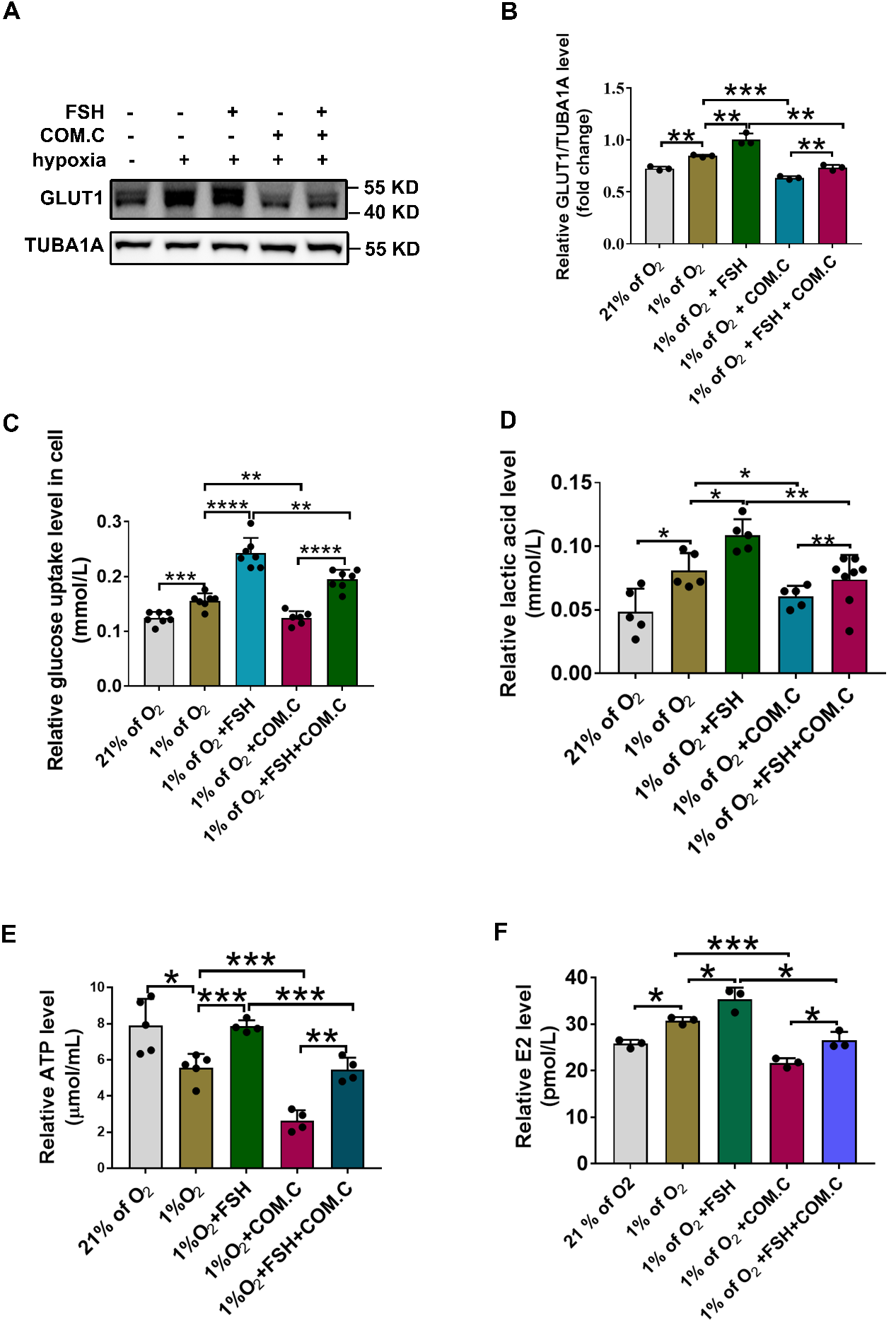


**Fig. S5**. Suppression of AMPK activity inhibits glycolytic metabolism and E2 production in FSH-treated GCs. **A**. GCs were cultured with normoxia hypoxia in the presence or absence of FSH for 24 h. For the inhibition of AMPK activity, COM.C were added 2 h prior to hypoxia exposure. The protein level of GLUT1 was determined using western blot. **B**. Quantitative analysis of GLUT1 levels. TUBA1A served as the control for loading. **C**. Glucose levels in GCs with indicated treatments. **D**. Determination of lactate levels in GCs with indicated treatments. **E**. Determination of ATP levels in GCs with indicated treatments. **F**. Measurement of E2 levels in culture medium of GCs with treatments as mentioned above. The corresponding data above are represented as mean ± SD. *P < 0.05; **P < 0.01; ***P < 0.001; ****P < 0.0001.


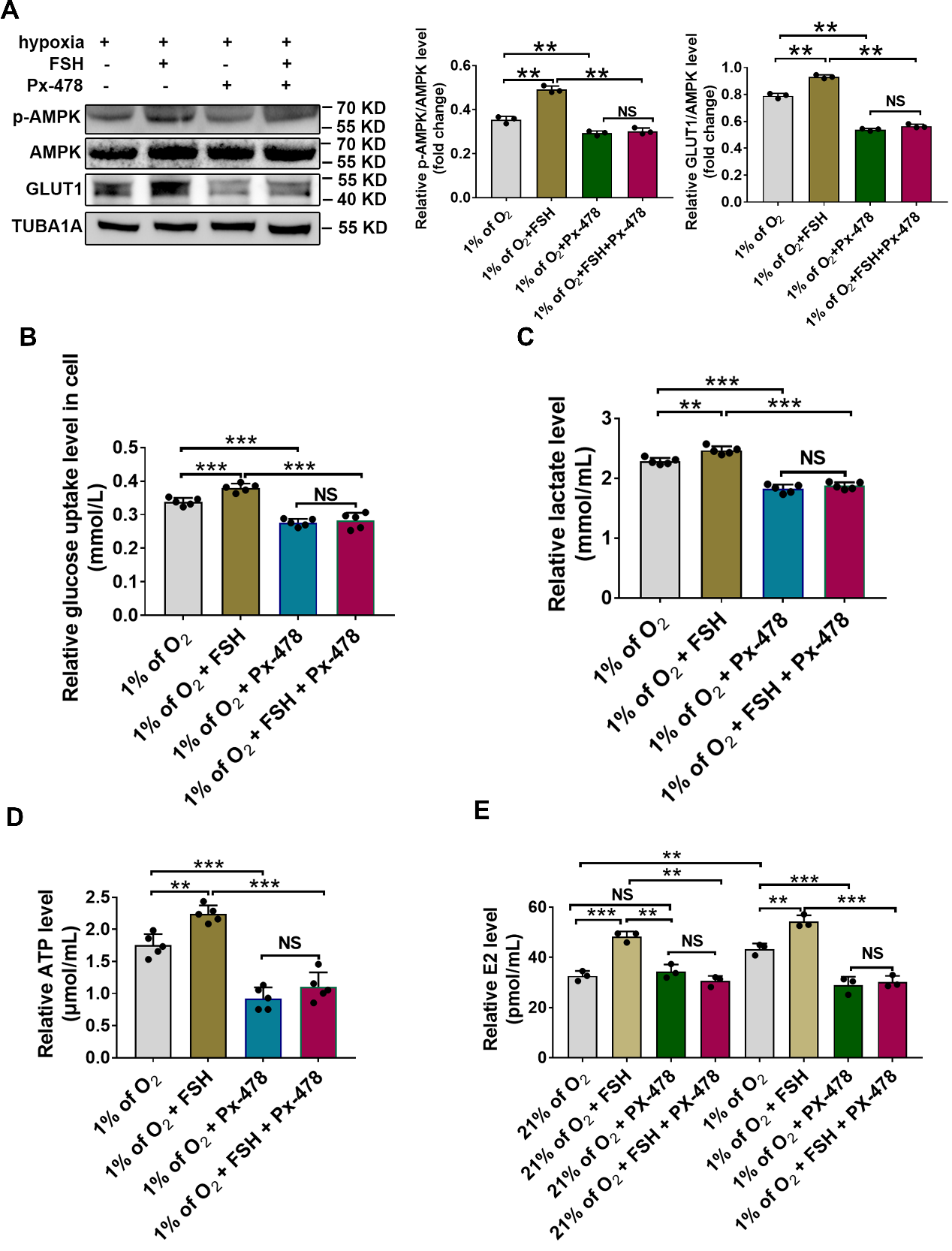


**Fig. S6.** FSH acts through HIF-1α to activate the AMPK/GLUT1 pathway in GCs exposed to hypoxia. **A**. GCs were cultured with hypoxia (1% of O_2_) and 3 IU/mL FSH for 24 h. PX-478 were added 2 h prior to hypoxia exposure. Cell lysates were then collected for determining protein levels of p-AMPK and GLUT1 using western blot. **B**. Glucose levels in GCs with indicated treatments. **C**. Determination of lactate levels in GCs with indicated treatments. **D**. Determination of ATP levels in GCs with indicated treatments. **E**. Determination of E2 levels in culture medium of GCs with treatments as mentioned above. The corresponding data above are represented as mean ± SD. *P < 0.05; **P < 0.01; ***P < 0.001

**Supplementary Table S1.** siRNA sequences

| *Scrambled siRNA* | Sense  （5'-3'） | UUCUCCGAACGUGUCACGUTT |
| --- | --- | --- |
|  | Antisense  （5'-3'） | ACGUGACACGUUCGGAGAATT |
| *Glut1 siRNA* | Sense  （5'-3'） | TGAAGGCGACCCCTGCTTA |
|  | Antisense  （5'-3'） | TAGGACCCCAGCGCATCTA |
| *AMPK-299*  *(AMPK siRNA1)* | Sense  （5'-3'） | GGCGAGCUAUUUGAUUAUATT |
|  | Antisense  （5'-3'） | UAUAAUCAAAUAGCUCGCCTT |
| *AMPK-792*  *(AMPK siRNA2)* | Sense  （5'-3'） | GGGAACAUGAAUGGUUUAATT |
|  | Antisense  （5'-3'） | UUAAACCAUUCAUGUUCCCTT |
| *AMPK-1453*  *(AMPK siRNA3)* | Sense  （5'-3'） | GGGCUCAGUUAGCAACUAUTT |
|  | Antisense  （5'-3'） | AUAGUUGCUAACUGAGCCCTT |
| *HIF-1α-960*  *(HIF-1α-siRNA1)* | Sense  （5'-3'） | GCCGCUCAAUUUAUGAAUATT |
|  | Antisense  （5'-3'） | UAUUCAUAAAUUGAGCGGCTT |
| *HIF-1α-1144*  *(HIF-1α-siRNA2)* | Sense  （5'-3'） | GUGCAUUGUAUGUGUAAAUTT |
|  | Antisense  （5'-3'） | AUUUACACAUACAAUGCACTT |
| *HIF-1α-1995*  *(HIF-1α-siRNA3)* | Sense  （5'-3'） | CCACCACUGACGAAUUAAATT |
|  | Antisense  （5'-3'） | UUUAAUUCGUCAGUGGUGGTT |

**Supplementary Table S2.** Primer sequences for qRT-PCR.

| *Glut1* | R | TGCTCCCCAGAAGGTGATCG |
| --- | --- | --- |
|  | F | CCGCAGTACACACCGATGAT |
| *Glut3* | F | TGAAGGCGACCCCTGCTTA |
|  | R | TAGGACCCCAGCGCATCTA |
